# Biotransformation of Carboxylic Acids to Alcohols: Characterization of *Thermoanaerobacter* Strain AK152 and 1-Propanol Production via Propionate Reduction

**DOI:** 10.3390/microorganisms8060945

**Published:** 2020-06-23

**Authors:** Sean Michael Scully, Johann Orlygsson

**Affiliations:** Faculty of Natural Resource Science, University of Akureyri, Borgir v. Nordurslod, 600 Akureyri, Iceland; scully@unak.is

**Keywords:** biocatalysis, extremophile, thermophile, fusel alcohols, carboxylic acids, volatile fatty acids, bioreduction

## Abstract

*Thermoanaerobacter* strains have recently gained interest because of their ability to convert short chain fatty acids to alcohols using actively growing cells. *Thermoanaerobacter thermohydrosulfuricus* strain AK152 was physiologically investigated for its ethanol and other alcohol formation. The temperature and pH optimum of the strain was 70 °C and pH 7.0 and the strain degraded a variety of compounds present in lignocellulosic biomass like monosaccharides, disaccharides, and starch. The strain is highly ethanologenic, producing up to 86% of the theoretical ethanol yield form hexoses. Strain AK152 was inhibited by relatively low initial substrate (30 mM) concentration, leading to inefficient degradation of glucose and levelling up of all end-product formation. The present study shows that the strain produces alcohols from most of the tested carboxylic acids, with the highest yields for propionate conversion to propanol (40.7%) with kinetic studies demonstrating that the maximum conversion happens within the first 48 h of fermentation. Various physiological tests were performed to maximize the acid conversion to the alcohol which reveals that the optimum pH for propionate conversion is pH 6.7 which affords a 57.3% conversion. Kinetic studies reveal that propionate conversion is rapid, achieving a maximum conversion within the first 48 h of fermentation. Finally, by using ^13^C NMR, it was shown that the addition of propionate indeed converted to propanol.

## 1. Introduction

The sustainable production of small molecular building blocks, such as alcohols is of great interest given the role that these chemicals serve in the production of more complex molecules. The hydration of petroleum-derived hydrocarbons presents a viable industrial route to the production of a wide variety of alcohols, while the fermentative production of alcohols is largely limited to simple alcohols. An attractive route to sustainable alcohol production from waste materials, such as through the bioreduction of inexpensive and abundant carboxylic acids presents a potential alternative for the production of higher chain alcohols.

The use of thermophiles for bioprocessing presents a number of advantages over traditional mesophilic bioprocessing organisms, such as higher growth rates, tolerance to many environmental extremes, and less risk of contamination from mesophilic organisms. The members of the genus of *Thermoanaerobacter*, all of which are thermophilic having temperature optima between 60 and 75 °C, have demonstrated diverse biotechnological potential including the production of bioethanol [1,2] and thermostable enzymes [3,4,5,6,7,8,9], notably secondary specific alcohol dehydrogenases (SADHs) producing chiral secondary alcohols from their corresponding ketones. *Thermoanaerobacter* strains have been isolated from a wide range of environments and possess a number of physiological features that make them potentially useful bioprocessing organisms. All members of *Thermoanaerobacter* have a broad substrate spectra, utilizing hexoses, pentoses, disaccharides, sugar alcohols such as mannitol [10], and polysaccharides, such as starch in addition to proteins and amino acids [11], as well as wide tolerance to fermentation conditions, including extremes of pH and temperature. One major limiting factor of wild type *Thermoanaerobacter* strains is that many show low tolerance for high (above 20 mM) initial substrate concentrations, although this can be overcome using other fermentation modes. A notable exception is *Thermoanaerobacter* strain J1, isolated from a hot spring in Iceland, which has high tolerance to high initial substrate loadings and producing ethanol as the dominant fermentation product [12].

Although the ability of auto- and lithotrophic *Clostridia,* such as the CO- and CO_2_-utalizing *Clostridium formicoacetium* [13], *C. autoethanogenum* [14], *C. carboxidivorans* [15,16], and *Morella thermoacetica* [17] to produce higher alcohols, such as 1-propanol has been long appreciated, the importance of the strains’ ability, within the class, to reduce carboxylic acids to their corresponding alcohols has only recently been recognized [18]. Recent work has also shown that some strains of *Thermoanaerobacter* can convert carboxylic acids to their corresponding primary alcohols in the presence of a source of reducing potential, such as glucose [19,20]. This is of potential utility as carboxylic acids are inexpensive, while their corresponding alcohols are useful chemical building blocks of greater value. Short-chain fatty acids (SCFAs) are ubiquitous in nature with acetic, propionate, and butyrate being common fermentation products by many anaerobes while branched-chain fatty acids such as 2-methyl-1-propionate (*iso*-butyrate), 2-methyl-1-butyrate, and 3-methyl-1-butyrate are common metabolites produced by the fermentation of branched-chain amino acids. Higher-order carboxylic acids (six or more carbon atoms) such as hexanoic acid and octanoic acid, which are commonly associated in goat milk and provides a characteristic odour.

The C3 alcohol 1-propanol (*n*-propanol, 1-PrOH) is a fusel alcohol and has received less attention than other biologically produced alcohols, such as ethanol and 1-butanol, despite its importance as a commodity chemical. 1-Propanol, like other alcohols, is an important solvent and chemical feedstock for propylene production, and as a raw material for the synthesis of esters and amines [21]. Given 1-propanol’s volatility, energy density, and high-octane number as well as it being less corrosive than ethanol, it is a potential alternative biofuel. The fermentative production of 1-propanol can occur via the Wood-Werkman pathway which is present in some *Clostridium* and *Propionibacterium* species [22,23,24] although examples of the production of 1-propanol via genetic modification have been reported [25,26,27,28].

*Thermoanaerobacter* strain AK152 was previously isolated from a hot spring in Iceland and is very closely related to *T. thermohydrosulfuricus* on the basis of its 16S RNA gene [29]. Strain AK152 was noted to produce branched-chain alcohols from branched-chain amino acids in much higher yields than *T. thermohydrosulfuricus* despite being very closely related and similar in many other regards [29]. The purpose of this work is to characterize *Thermoanaerobacter* strain AK152 and investigate its ability to rapidly convert short-chain carboxylic acids to their corresponding alcohols and move towards optimizing reaction conditions, in order to improve the yields of the resultant primary alcohols. As this strain has been previously demonstrated to produce branched chain alcohols from some amino acids under electron scavenging conditions, a particular emphasis was placed on the impact of culture conditions on carboxylic acid reduction, using propionate as a model compound to maximize 1-propanol yields.

## 2. Materials and Methods

### 2.1. General Methods and Cultivation

All materials were acquired from Sigma Aldrich, with the exception of ^13^C-labeled carboxylic acids which were acquired from Cambridge Isotope Laboratories (Tewksbury, MA). Nitrogen gas contained less than 5 ppm of O_2_. *Thermoanaerobacter* strain AK152 (formerly called strain H1), which was isolated from a hot spring in Iceland as previously described [12], was re-activated from a glycerol (30% *v/v*) freezer stock stored at –20 °C. *Thermoanaerobacter thermohydrosulfuricus* (DSM 567) was obtained from Leibniz-Institute DSMZ-Deutsche Sammlung von Mikroorganismen und Zellkulturen GmbH.

Cultivations were carried out using basal mineral medium (BM medium) which was prepared, as previously described [10]. All stock solutions were syringe filtered through 0.22 µm syringe filters into sterile nitrogen flushed bottles with the exception of polymeric substrates which were added to the medium prior to autoclaving. Stock solutions of carboxylic acids were titrated to pH 7.0 ± 0.3 and syringe filtered into nitrogen flushed bottles. Cultivations were performed in either, serum bottles or Hungate tubes, without agitation at 65 °C at pH 7 and a liquid-gas phase ratio of 1:1 (see below in Section 2.3) for a period of 5 days unless stated otherwise. The inoculation volume was 2% *v/v* taken from cultures in the late exponential growth phase cultivation on glucose (20 mM). All experiments were conducted in triplicate unless stated otherwise.

### 2.2. Characterization and Substrate Spectra

The determination of temperature range and optimum was determined in BM medium (pH 7.0) in 117.5 mL serum bottles. The determination of pH range and optimum was performed at 70 °C from pH 4 to 9. Samples were periodically withdrawn and the optical density analyzed. The utilization of carbon sources was performed in 25 mL serum bottles at 65 °C at pH 7 and a liquid-gas (L-G) ratio of 1.0 containing 20 mM of substrate with the exception of solid polymeric substrates, which were provided as a 2% *w/v* solution. End products were quantified after 5 days of fermentation. API ZYM strips (BioMérieux, France) were used to determine the presence of specific enzyme chemistries accordance with the manufacturer’s directions except that the incubation was performed in a humidified bag at 65 °C for 4 h prior to reagent addition; API ZYM strips were performed in duplicate.

### 2.3. Influence of Initial Glucose and Liquid-gas Phase Ratio

The influence of L-G ratio was evaluated in 117.5 mL serum bottles with varying liquid volumes to yield L-G ratios of 0.017, 0.044, 0.093, 0.34, 1.04, and 3.27. Glucose concentration applied was 20 mM. Similarly, the influence of initial glucose concentrations were evaluated between concentrations of 5 and 400 mM at an L-G ratio of 1.00; fermentation end product were analyzed after 5 days of incubation.

### 2.4. Reduction of Carboxylic Acids Using the Glucose as a Source of Reducing Potential

*Thermoanaerobacter* strain AK152 was cultivated in Hungate tubes (18 × 150 mm) containing glucose (20 mM) and an exogenously added carboxylic acid at a concentration of 20 mM. The carboxylic acids examined included formate, acetate, propionate, 2-methyl-1-propionate, 1-butyrate, 1-pentanoate, 2-methyl-1-butyrate, 3-methyl-1-butyrate, 1-hexanoate, 1-heptanoate, and 1-octanoate. Control bottles contained only yeast extract or glucose (20 mM). Fermentation products were quantified after 5 days of fermentation.

### 2.5. Influence of Initial Carboxylic Acid Concentration

The influence of carboxylic acid concentration on glucose fermentation (20 mM) was evaluated between 10 and 100 mM. End products were quantified after 5 days of fermentation.

### 2.6. Kinetic Study of Carboxylic Acid Reduction by Thermoanaerobacter Strain AK152

To evaluate the effect of exogenously added carboxylic acid on glucose fermentation, strain AK152, three concentrations (25, 50, and 100 mM) of acetate, propionate, or butyrate were added to in experimental bottles containing 20 mM of glucose. Fermentations were performed in serum bottles (125 mL). Samples were withdrawn after 12, 24, 36, and 168 h for analysis.

### 2.7. ^13^C NMR Experiments

^13^C-labelled carboxylic acids were obtained from Cambridge Isotope Laboratories (MA, USA). The strain was cultivated in the presence of ^13^C1-labeled carboxylic acids and glucose (20 mM) for 5 days. 1 mL of fermentation broth was then analyzed using a Bruker AV400 after spiking with 0.3 mL of D_2_O to obtain signal lock as previously described [30]

### 2.8. Analytical Methods

The optical density of cultures was measured using a Shimadzu UV-1800 UV-Visible spectrophotometer at 600 nm with a path length of 1 cm. Hydrogen was quantified using an AutoSystem XL (Perkin Elmer) gas chromatograph equipped with a TCD, as previously described [31]. Cell-free extracts were prepared by centrifugation at 13,000 rpm for 3 min and were used for all subsequent analysis. Volatile end products were analysed using a Clarus 580 (Perkin Elmer) gas chromatograph equipped with a FID as described by [31].

Lactate was quantified colorimetrically according to the method of Taylor [32] with the following modifications described by earlier [33]; 300 µL of concentrated sulphuric acid was added to 50 µL of sample in an Eppendorf tube. The sample was incubated in a boiling water bath for 10 min and then cooled to room temperature. After cooling, 5 µL of 4% *w/v* copper (II) sulphate and 10 µL of 1.5% (*w/v*) *p*-phenyl phenol (in 95% ethanol) were added. The samples were vortexed and incubated at room temperature for 30 min. After incubation they were read at 580 nm against a BM medium blank. Glucose was quantified using the 3,5-dinitrosalicylic acid (DNS) method of [34] with modifications. 100 µL of a sample and 100 µL of DNS reagent (1.0 g of 3,5-dinitrosalicylic acid, 5.0 g of sodium sulfite, 4 g of sodium hydroxide dissolved in 100 mL of dH_2_O) was added to a microtiter plate and incubated at 90 °C for 20 min. After cooling for 5 min, 33 µL of 40% *w/v* tartaric acid solution and the plate mixed (~750 rpm for 30 s) prior to reading at 580 nm on a Bioscreen C against a water blank.

## 3. Results

### 3.1. Strain Characterization

**Figure 1:**
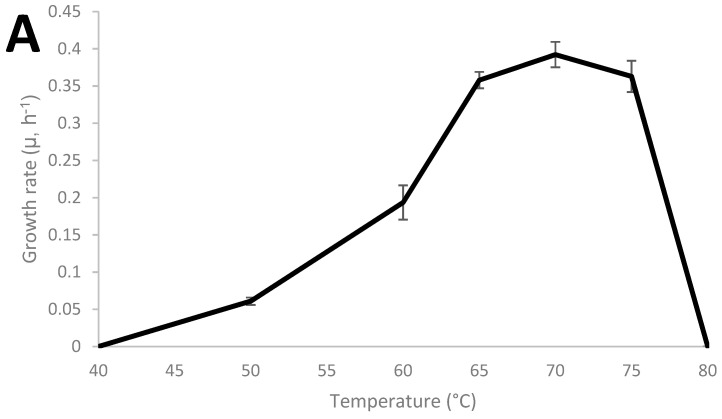

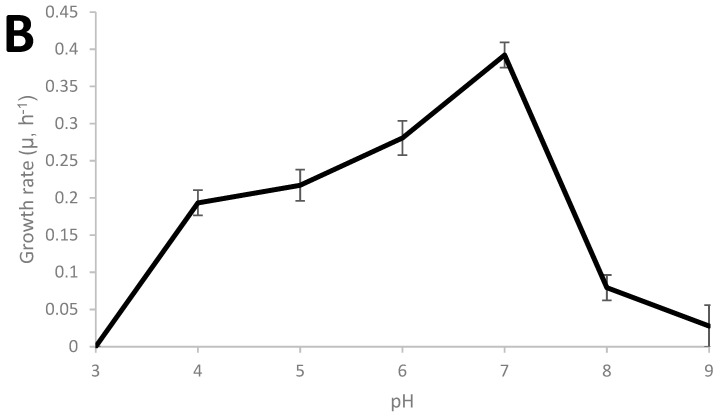
Growth characteristics of *Thermoanaerobacter* strain AK152; influence of cultivation temperature at pH 7.0 (**A**) and pH at 70 °C (**B**).

*Thermoanaerobacter* strain AK152 has 99.5% similarity to *Thermoanaerobacter thermohydrosulfuricus* based on sequencing of the 16S RNA gene (KR007666), as previously described [29]. The growth characteristics of the strain were characterized and the strain was found to grow from 45 °C to 75 °C with an optimum of 70 °C and a pH range from 4 to 8 with an optimum of pH 7 as shown in Figure 1A,B, respectively. Under the strain’s optimum growth conditions (70 °C, pH 7.0), a doubling time of 1.76 h (106 min) was observed.

**Figure 2:**
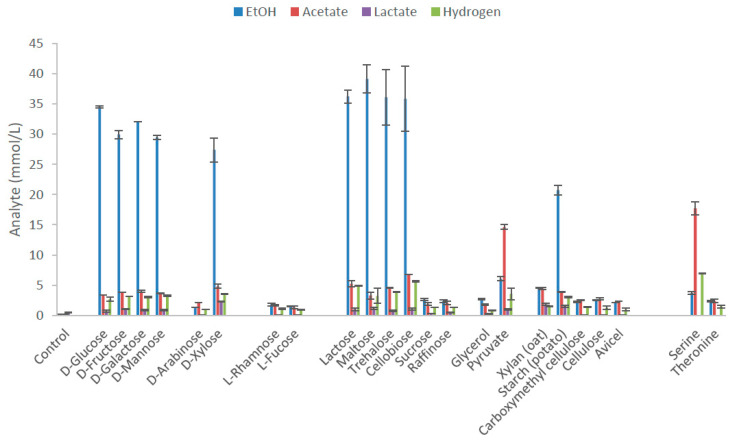
End product formation after 5 days by *Thermoanaerobacter* strain AK152 for selected carbon sources (65 °C, pH 7.0) at a concentration of 20 mM with the exception of polymeric substrates which were provided at 0.2% *w/v*. Values represent average of triplicate fermentations with standard deviation presented at error bars.

*Thermoanaerobacter* strain AK152 degrades a wide range of substrates including hexoses, xylose and several disaccharides. The main end-product from substrates, with the exception of serine and pyruvate, was ethanol with yields on various hexoses between 73.6 and 86.2% of the theoretical yield and 82.2% on xylose (Figure 2, Supplementary Table S1). Other observed end-products included acetate and lactate although with yields lower than 12.5% of theoretical yields (5 mM). The strain did not utilize L-rhamnose, L-fucose, sucrose, raffinose, xylan, CMC, cellulose, avicel, and threonine. The strain also possess C4 esterase and acid phosphate activity (Supplementary Table S2). Serine and pyruvate fermentation produced acetate as the dominant end product.

### 3.2. Influence of Culture Conditions

**Figure 3:**
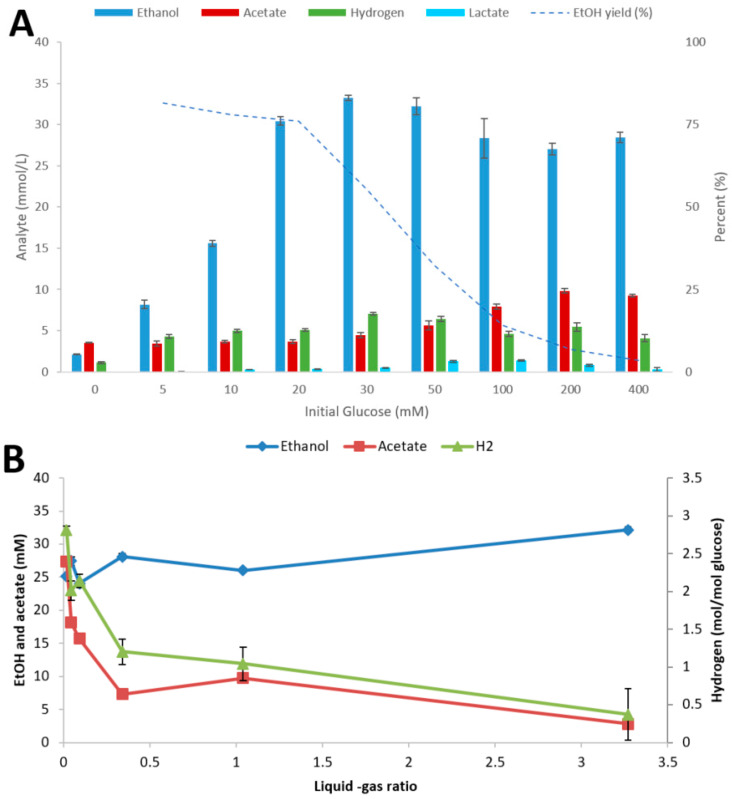
Influence of initial glucose concentration (**A**) and liquid-gas phase ratio (using 20 mM glucose) (**B**) on fermentation by *Thermoanaerobacter* strain AK152. Fermentations were performed at 65 °C and pH 7.0 with end products being quantified after 5 days. Values represent average ± standard deviation (*n* = 3).

To investigate the influence of culture conditions on the fermentation performance of strain AK152, the strain was cultivated at various initial glucose concentrations and L-G ratios, as shown in Figure 3A,B, respectively.

At initial glucose concentrations below 30 mM, the strain completely degraded the sugar with the main end-product being ethanol (Figure 3A). At higher substrate loadings strong inhibition occurred with almost no glucose degradation at the highest loadings used (400 mM). Different L-G ratios had some impact on the end product profiles of *Thermoanaerobacter* strain AK152 with ethanol concentrations highest at a high L-G ratio and acetate and hydrogen lowest and vice versa (Figure 3B). Thus, the ratio of ethanol from low to high L-G ratio increased by 37%, whereas acetate concentrations decreased by a factor of five with increasing L-G ratio.

### 3.3. Carboxylic Acids Reduction by Thermoanaerobacter Strain AK152

**Figure 4:**
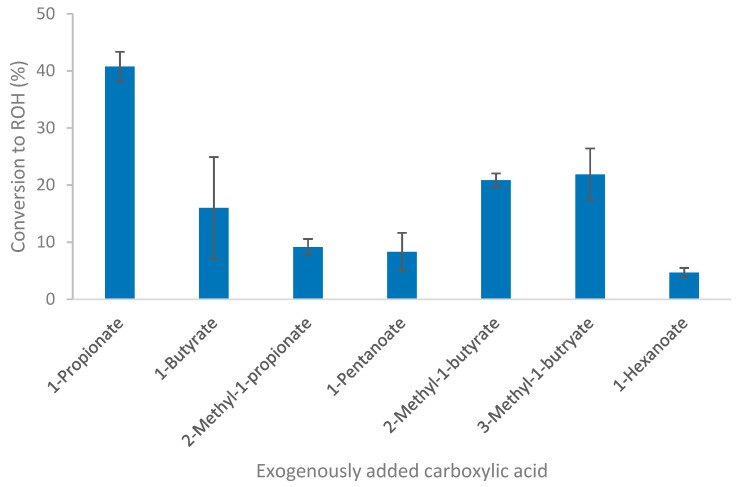
Conversion of short-chain fatty acids by *Thermoanaerobacter* strain AK152 using glucose (20 mM) as a source of reducing potential. Values represent average ± standard deviation (*n* = 3).

The ability of *Thermoanaerobacter* strain AK152 to reduce C1 to C8 carboxylic acids was evaluated in batch culture using glucose as a source of reducing potential. The fermentation profiles of strain AK152 on glucose-containing medium supplemented with carboxylic acids confirm that the strain could reduce C3 to C6 carboxylic acids to their corresponding alcohols, as shown in Figure 4. Conversion yields were between 4.7 and 40.7%, with higher conversions generally being observed for smaller carboxylic acids. A notable exception of this trend occurred in the case of the reduction of 2- and 3-methyl-1-butyrate for which conversion of around 20% were obtained. The highest observed conversion, 40.7%, was that of propionate to 1-propanol (8.08 mM). Only 4.7% of the hexanoate was converted to 1-hexanol to afford a concentration of less than 1.0 mM. While formate and acetate were examined, there was negligible evidence of methanol formation (from formate) and ethanol formation from acetate reduction was masked by background ethanol production from glucose fermentation.

It should be noted that the addition of formate caused a decrease in the amount of acetate and hydrogen formed as compared to glucose controls. However, the addition of heptanoic acid and octanoic acid caused ethanol formation to increase to near theoretical yields although none of the corresponding alcohols (1-heptanol or 1-octanol) were observed. The ability of carboxylic acid reduction by *Thermoanaerobacter thermohydrosulfuricus* was also examined but did not yield any of the corresponding alcohols.

### 3.4. Influence of Culture Conditions on Carboxylic acid Reduction

**Figure 5:**
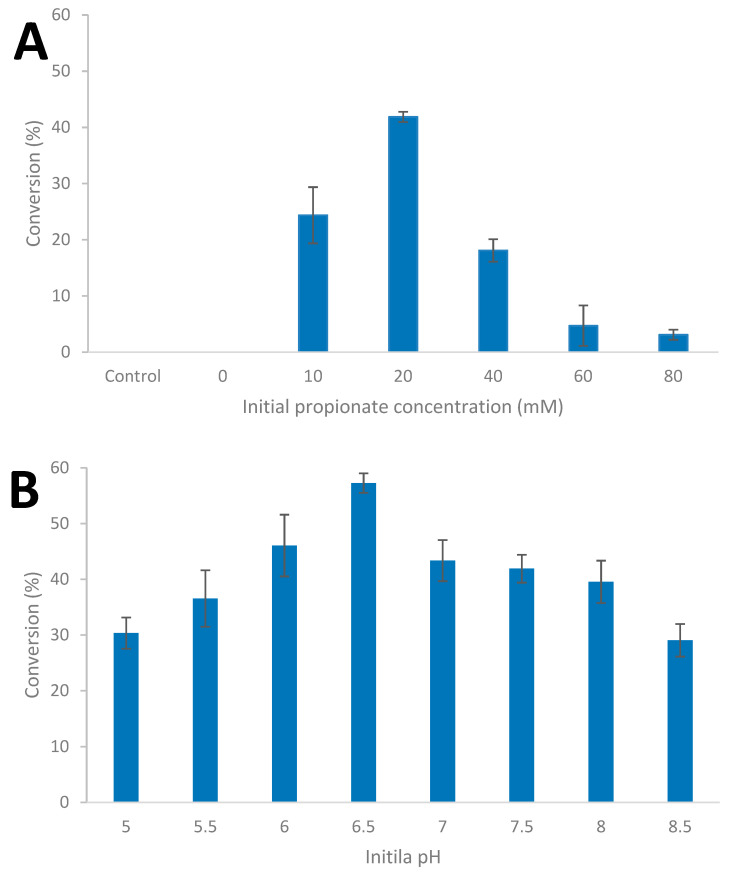
Influence of initial propionate concentration at 65 °C and pH 7.0; (**A**) and initial pH with 20 mM propionate concentration; (**B**) during glucose (20 mM) fermentation by *Thermoanaerobacter* strain AK152 (65 °C. Values represent the average of triplicates with standard deviation presented as error bars.

In order to evaluate the impact of culture conditions on carboxylic acid reduction, the influence of increasing initial concentration of the carboxylic acid, and initial pH, were evaluated in batch culture; it should be noted that the BM medium used contains 50 mM of phosphate buffer. The strain was cultivated on glucose (20 mM) supplemented with between 10 and 80 mM of carboxylic acid as shown for propionate (Figure 5A) and the influence of initial pH between 4.0 and 8.5 (Figure 5B, Table S4).

Increasing the initial concentration of propionate did not result in an increase in 1-propanol formation; the conversion of propionate ranged from 41.9% at an initial concentration of 20 mM of propionate to 3.1% conversation at an initial concentration of 80 mM. With the addition of propionate, end product formation shifted towards acetate with less ethanol being formed (Supplementary Table S3). The amount of glucose utilized by the strain decreased to around 90% at an initial propionate concentration of 80 mM (Supplementary Table S3). With respect to the impact of initial pH, the percent of propionate reduced to 1-propanol ranged from approximately 30% at pH 4 and 8.5 to a maximum of 57.3% at pH 6.5.

### 3.5. Kinetic Experiments

**Figure 6:**
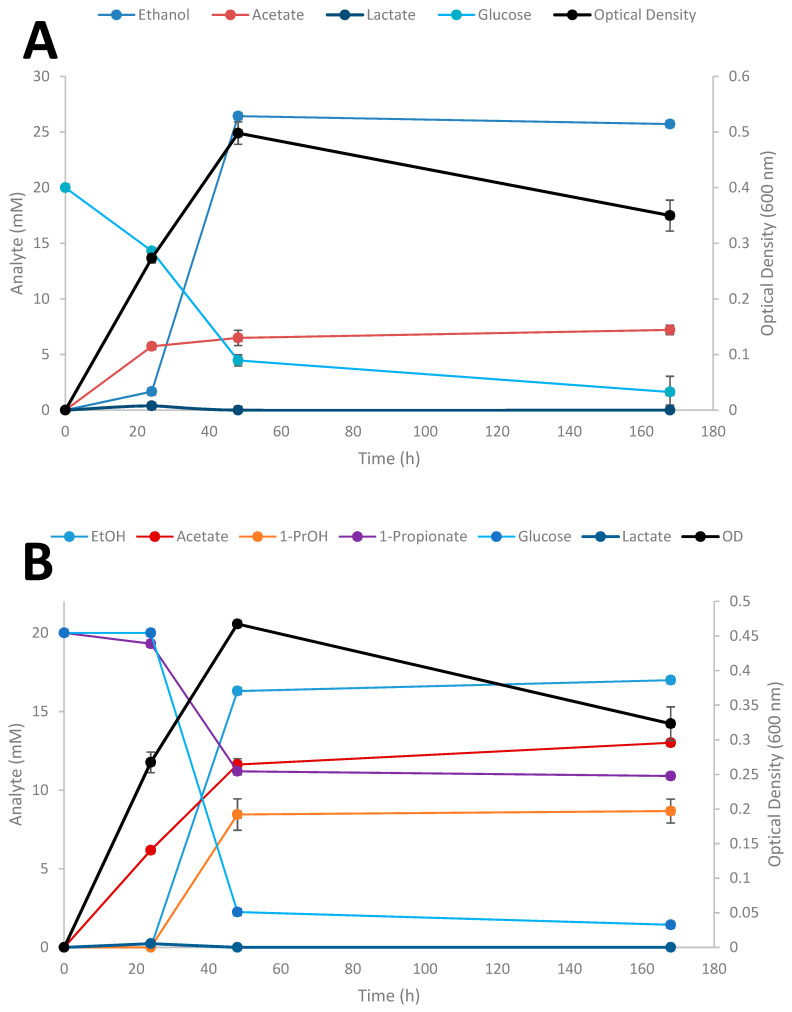

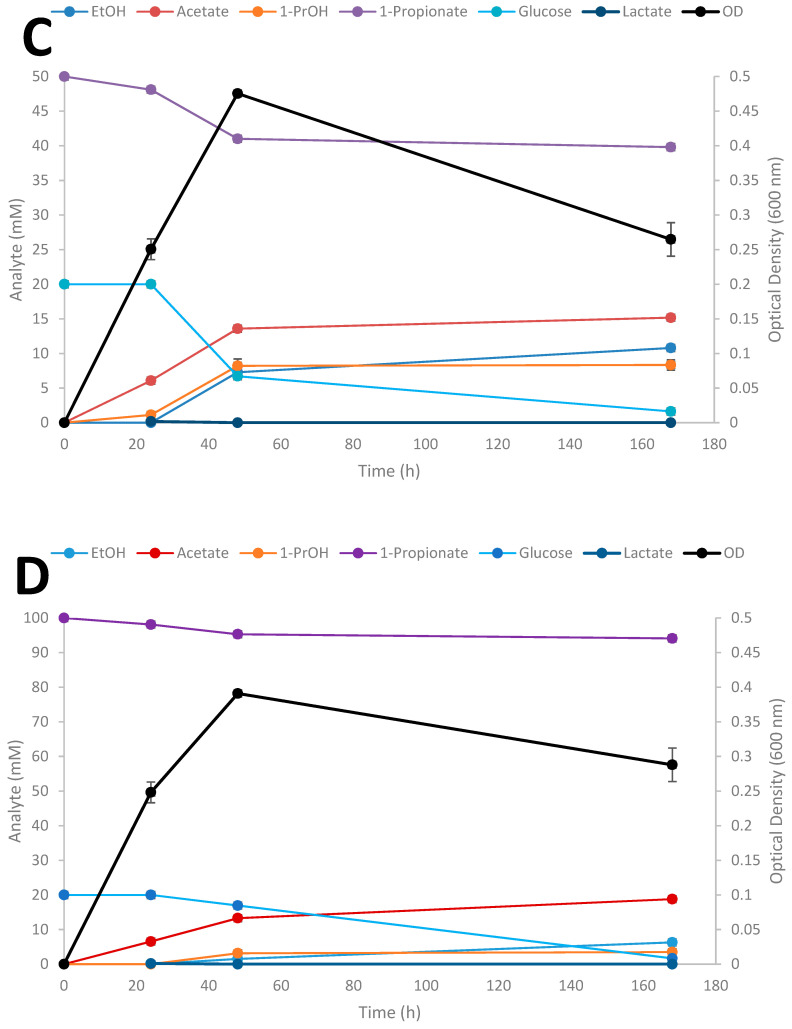
Fermentation of 20 mM glucose (**A**), 20 mM glucose + 20 mM propionate (**B**), 20 mM glucose + 50 mM propionate (**C**), 20 mM glucose + 100 mM (**D**) by *Thermoanaerobacter* strain AK152. Values represent the average of triplicates ± standard deviation.

Time-course studies on the conversion of SCFAs in the presence of glucose by *Thermoanaerobacter* strain AK152 was evaluated over a 7 day period for propionate at three different initial concentrations of the acids (Figure 6A–D); results for the influence of exogenously added acetate and butyrate are provided in supplemental material (Supplementary Figures S1 and S2, respectively).

When glucose is provided as the sole substrate, strain AK152 reaches its maximum ethanol titer (26.4 mM) within 48 h of fermentation having consumed 15.5 mM of glucose (77.5% consumed) (Figure 6A). In contrast, when propionate (20 mM) is added, more glucose is consumed (17.8 mM or 89.0% consumption) with propionate being converted to 1-propanol (8.5 mM) and a decrease in ethanol formation (16.3 mM) as compared with fermentation on glucose alone (Figure 6B). Similarly, when a higher propionate concentration (50 mM) is added to glucose fermentation, a similar amount of 1-propanol (8.2 mM) is achieved after 48 h but ethanol concentrations were much lower or only 7.3 mM (Figure 6C). At the highest initial addition of propionate (100 mM) less 1-propanol (3.2 mM) and ethanol (6.3 mM) were produced (Figure 6D). At the increased propionate loadings of 50 and 100 mM, glucose utilization decreases, and is only 66.4 and 15.4% of the glucose after 48 h fermentation, respectively. Similar experiments using acetate and butyrate were also performed showing similar trends as observed for propionate reduction (Supplementary Figures S1 and S2, respectively).

### 3.6. NMR with Thermoanaerobacter strain AK152

**Figure 7:**
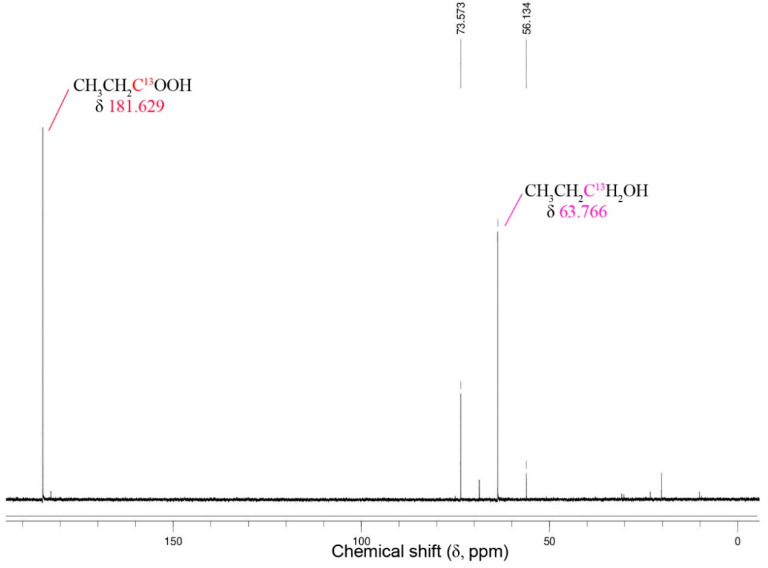
^13^C NMR spectra for the reduction of ^13^C1-labeled propionate by *Thermoanaerobacter* strain AK152 after 5 days of fermentation with glucose (20 mM) at 65 °C, pH 7.0.

To demonstrate that the 1-propanol produced by 1-propionate reduction is not a novel fermentation products, the use of ^13^C-labeled carboxylic acid were added to an active culture of strain AK152 with glucose as the carbon and energy source (Figure 7).

A peak attributable to the ^13^C-labeled propionate and its corresponding alcohol are apparent at 181.6 ppm, and 73.7 ppm, respectively. The relative intensity suggests that about 40% of the ^13^C-labeled propionate was reduced to 1-propanol. Similar results for ^13^C1-labeled acetate and 1-butyrate were obtained (Supplementary Figure S3).

## 4. Discussion

*Thermoanaerobacter* strain AK152 is closely related to *Thermoanaerobacter thermohydrosulfuricus* and has a similar growth range as a function of temperature (50/70/75 as compared to 37/70/78, respectively) and pH (4.0/7.0/8.0 vs. 5.5/6.9–7.5/9.2, respectively) although AK152 is less tolerant to highly alkaline conditions [7]. Like many *Thermoanaerobacter* species, strain AK152 utilizes a range of hexoses, pentoses, di- and polysaccharides (Figure 2). Compared to many closely related *Thermoanaerobacter* strain, AK152 is highly ethanologenic producing around 75% of the theoretical ethanol yield from 20 mM of glucose. Interestingly, AK152 does not utilize sucrose, which is an otherwise ubiquitous feature of *Thermoanaerobacter* species. This may be explained by the apparent lack of α-glucosidase activity observed on the API ZYM strips. Furthermore, it is noteworthy that the strain does not utilize arabinose, a pentose found in some forms of hemicelluloses. The strain weakly utilizes xylan, even though the end products formed are still less than other carbon sources. The presence of phosphatases revealed by API ZYM strips likely aid the strain in acquiring inorganic and organic phosphates from its native habitat. Similar to other *Thermoanaerobacter* species, strain AK152 possess short-chain esterase and acid phosphatase activity but lacks the alkaline phosphatase activity (Supplementary Table S2) seen among other closely related *Thermoanaerobacter* species.

*Thermoanaerobacter* strain AK152 has previously been shown to produce a mixture of branched-chain fatty acids and branched-chain alcohols during amino acid degradation [29] and can also degrade serine and pyruvate without an electron acceptor. Recent investigations have demonstrated that the ability to reduce carboxylic acids to their corresponding primary alcohols is present among some *Thermoanaerobacter* strains such as *T. pseudoethanolicus* [19,20]. Similar to *T. pseduoethanolicus*, strain AK152 can convert C3-C6 carbxoylic acids to their corresponding alcohols although strain AK152 has a higher preference for propioante reduction. Recent work with *Thermoanaerobacter* strain AK85 demonstrated that its ability to produce branched-chain alcohols from the corresponding amino acid was likely due to the strain´s ability to reduce carboxylic acids, such as 3-methyl-1-butyrate, to the corresponding alcohol in the presence of reducing equivalents [30]. Based on the results reported here, it is very likely that carboxylic acid reduction may indeed be responsible for strain AK152′s ability to produce branched-chain alcohols from branched-chain amino acids. While the conversion yields of carboxylic acids to their corresponding alcohols by *Thermoanaerobacter* strain AK152 are lower than those reported for *T. pseudoethanolicus*, the prescence of this chemistry suggests that this capaiblity may be a common feature among the genus, although, the closely related *Thermoanaerobacter thermohydrosulfuricus* lacks this capability. It is known that carboxydotrophic bacteria like *Clostridium ljungdahlii* and "*Clostridium ragsdalei”* can convert fatty acids to alcohols using syngas as reducing power [35,36]. Whether strain AK152 can utilize hydrogen as a reduing power instead of glucose remains to be determined.

In the context of 1-PrOH production produced by other means, such as fermenation from wild-type and genetically modified organisms, the ability of strain AK152 is modest but presents a novel route to 1-propanol and other primary alcohols from their corresponding carboxylic acids. Most of the literature on the fermentation production of 1-propanol among *Clostridia* is limited, typically with only trace quantities being reported (Table 1). As such, the yields reported here from the bioreduction of propinate fall short of the 3.5 g/L achieved using genetically modified *E. coli* [25] but are higher than those previously reported for propioante reduction by *T. pseudoethanolicus*. While the conversion efficiences of *Thermoanaerobacter* strains are lower than some of the CO- utilizing *Clostridium* strains that have been reported [35,36], it is noteworthy that *Thermoanaerobacter* strain AK152 achieves its maximum conversion within 48 h (Figure 6B) rather than over several days in cases where CO is used as a source of reducing potential, which may be due to the limitations of gas solubility and mass transfer. Achieving rapid carboxylic acid reduction with higher conversion efficiencies using strain AK152 or other *Thermoanaerobacter* strains might be possible using fed-batch fermentations or by using a biphasic system or gas stripping for continuous alcohol removal.

To study the impact of culture conditions on carboxylic acid reduction in more detail, 1-propionate was selected as a model carboxylic acid. Like other thermophilic anaerobes, *Thermoanaerobacter* strain AK152 is sensitive to elevated initial substrate concentrations with a decrease in substrate utilization being profound above 30 mM of glucose (Figure 3A). Unlike *Thermoanaerobacter* strain AK68 [37], AK152 is not particularly sensitive to changes in L-G ratio, in that glucose utilization did not drop or end product formation shift. That said, ethanol yields did increase slightly with increasing L-G ratios with a corresponding decrease in acetate and hydrogen production which suggests that the partial pressure of hydrogen may play a minor role in directing end product profiles (Figure 3B). There is a tolerance to carboxylic acids, such as 1-propionate, initial concentrations of higher loadings of 1-propionate. Howefver, this resulted in strong inhibition as evidenced by poor glucose utilization and a leveling off of ethanol formation around 10 mM at concentarionst greater than 60 mM of 1-propioante (Supplementary Table S3). Furthermore, the amount of 1-propionate converted to 1-propanol was higher at lower initial propionate concentrations which suggests that the ratio of the source of reducing potential to carboxylic acid may be of importance (Figure 5A).

It is known that “*Clostridium ragsdalei”* uses an aldehyde:ferredoxin oxidoreductase-like enzyme to reduce *n*-fatty acids to the aldehyde intermediates in a reaction that requires ferredoxin and exogenous carbon monoxide. Prior work has demonstrated that both aldehyde oxidoreductase and alcohol dehydrogenases are active in *Thermoanaerobacter* strains demonstrating carboxylic acid reduction [19,30] and the same is likely to be the case with strain AK152 despite the apparent differences in substrate preference. Examinations of carboxylic acid reducing autotrophic “*Clostridium ragsdalei”* has demonstrated that, beyond requiring an AOR, a ferredoxin is required to direct reducing potential from CO to the aldehyde intermediate^36^. *Thermoanaerobacter* strains use ferredoxin [38] although the movement of reducing potential is more complex, often requiring both primary and secondary ADHs with NAD, and NADP, respectively. In this case, the nature of the metabolic intermediate in carboxylic acid reduction remains unclear and may be either an aldehyde or an acyl-CoA although the ^13^C NMR spectra obtained does not implicate the presence of either in detectable quantities. It is likely that such an intermediate is short-lived and further work to identify the role of either or both intermediates is required. Given the differences between strain AK152 and *T. hydrosulfuricus* in terms of their ability to reduce carboxylic acids, the whole genomes of both organisms will be compared in future work.

While the range of substrate investigated here is largely limited to straight-chain alkyl carboxylic acids, the ability to reduce more sterically bulky carboxylic acids, such as phenylacetic acid or more branched carboxylic acids such as 4-methyl-pentanoic acid, could be a useful route in producing larger alcohols in an aqueous system from renewable sources of reducing potential, such as biomass hydrolysates or waste polyalcohols such as glycerol.

Compared to the previously described reduction of ^13^C1-labeled carboxylic acids by *Thermoanaerobacter pseudoethanolicus*, the relative peak intensities attributable to the formation of the corresponding primary alcohol are much lower. This is in good general agreement with the results obtained from the analysis of end products from the other experiments with strain AK152. It should be noted that the appearance of unidentified peaks produced during the reduction of ^13^C1 acetate and ^13^C1-propionate differs from the spectra obtained from *T. pseudoethanolicus* and may suggest that there is an accumulation of ^13^C-labeled intermediates or the production of other end products entirely. Following the reduction ^13^C-labeled carboxylic acids with strain AK152 may shed further light on this as would further analysis using mass spectrometry.

The vast differences in substrate specificity of carboxylic acids being reduced between *Thermoanaerobacter* strains may suggest that other strains have different substrate preferences, as evidenced by strain AK152’s preference for propionate reduction and its poor ability to reduce larger carboxylic acids, such as hexanoate. This stands in contrast to previously data from of *T. pseudoethanolicus* which not only reduces propionate, but has a clear preference for the reduction of 2-methyl-1-propionate and converts a modest amount of hexanoate to 1-hexanol [20] while other strains such as *T. hydrosulfuricus* (this study) and *T. mathranii* [19] lack the ability to reduce carboxylic acids entirely. Differences in the structure of the active sites of the enzymes involved are likely responsible for the differences in substrate preferences, as observed among the TSADHs that have been examined previously [39,40]. It should be noted, however, that another explanation may be that the alcohols produced by carboxylic acid reduction are very likely to be more inhibitory than ethanol [41,42] which may be partially responsible for the lower conversions of C4 and higher carboxylic acids to their corresponding alcohols.

The propionate conversion yields here could potentially be improved to maximize the amounts of 1-propanol produced. A better approach may involve the use of pre-cultivated whole-cells. The role of the ratio of reducing potential to carboxylic acid warrants further investigation as does the use of other fermentation modes, such as fed-batch or continuous culture, as a means of circumventing the observed inhibition from glucose at concentrations greater than 30 mM. That said, the impact of alcohol inhibition, particularly from higher order alcohols, requires further work and may need the use of a biphasic system or gas stripping to prevent the accumulation of cytotoxic primary alcohols. Other electron donors, such as biomass hydrolysates or waste streams rich in reducing potential, may make viable alternative sources of reducing potential. Given the ubiquity of carboxylic acids in waste streams (agricultural residues, anaerobic digestate, aquaculture runoff, mining waste, etc), the conversion of these carboxylate-rich waste streams with *Thermoanaerobacter* strains may be a worthwhile route to the valorization of otherwise low-value and problematic waste streams.

The capability of *Thermoanaerobacter* species to reduce carboxylic acids in the presence of glucose could present a novel route to the production of higher-order alcohols from inexpensive feedstocks, e.g., lignocellulose or waste lactose from whey production, using the reducing power of glucose or other inexpensive materials. *Thermoanaerobacterium* strains have been noted to rapidly reduce ^13^C-labeled exogenously added acetate to ethanol via an acetyl-CoA intermediate [45]. Carboxylic acid reassimilation may provide insight as to why some *Thermoanaerobacterium* and *Thermoanaerobacter* strains are such efficient ethanol producers during glucose fermentation.

## 5. Conclusions

*Thermoanaerobacter* strain AK152 is a thermophilic anaerobe with the ability to grow using a broad range of temperatures and pH (50/70/75 °C and pH 4.0/7.0/8.0) with the ability to utilize a wide range of carbohydrates. While the strain has limitations such as its sensitivity to elevated glucose concentrations, it possess the ability to reduce C2 to C6 carboxylic acids to their corresponding primary alcohols using glucose as a source of reducing potential. The highest overall conversions of carboxylic acids to alcohols were observed for propionate to propanol (40.7% conversion) and for 2- and 3-methyl-1-butyrate, which gave 20.9 and 21.9% conversions, respectively. Culture conditions can influence the efficacy of propionate reduction with elevated initial 1-propionate causing inhibition at higher concentrations, while an initial pH of 6.5 results in optimum reduction of 1-propionate to 1-propanol (57.3% conversion). The conversion of 1-propionate to 1-propanol is rapid, occurring within the first 48 h of glucose fermentation. The use of ^13^C1-labeled carboxylic acids indeed confirms that the source of the observed primary alcohols is from reduction of the exogenously added carboxylic acid.

## Figures and Tables

**Table 1:** Yields of 1-Propanol from selected organisms; red- reduction; GE-genetically engineered; Glu- glucose; W-W – Wood-Werkman pathway, Suc-succinate pathway, Cit-Citramalate pathway, Thr- Threonine pathway, 1,2-PD- 1,2-propanediol pathway.

Organism	Pathway	Substrate	1-PrOH Titer	Conversion (%)	Reference
*Thermoanaerobacter* strain AK152	RCOOH red	Glu (20 mM) +propionate (20 mM), pH 6.5	691 mg/L (11.5 mM)	57.3%	This study
*Thermoanaerobacter* strain X514	RCOOH red, cell suspension	Glu (25 mM) + propionate (50 mM)	1.5 g/L (25 mM)	50%	[19]
*T. pseudoethanolicus*	RCOOH red	Glu (20 mM) +propionate (20 mM)	397 mg/L (11.5 mM)	33%	[20]
*T. brockii* subsp. *finnii*	RCOOH red, cell suspension	Glu (25 mM) + propionate (50 mM)	1.3 g/mL (21 mM)	42%	[19]
*C. ljungdahlii* ERI-2	RCOOH red	CO + propionate (15 mM)	627 mg/mL (10.4 mM)	69.4%	[36]
*“C. ragsdalei”*	RCOOH red	CO + propionate (15 mM)	451 mg/L (7.5 mM)	30%	[36]
*“C. ragsdalei”*	RCOOH red	CO + propionate (30 mM)	1.74 g/L (29 mM)	97%	[35]
*P. acidipropionicum*	W-W	Glycerol (20 g/L)	<2.0 g/L	N/A	[43]
*P. acidipropionicum*	W-W	Glu (20 g/L)	<1.0 g/L	N/A	[43]
*P. freudenreichii*	W-W	Glycerol (20 g/L)	<2.0 g/L	N/A	[43]
*P. freudenreichii*	W-W	Glu (20 g/L)	<1.0 g/L	N/A	[43]
*E. coli*	GE (Suc)	Glu (20 g/L) + succinate (4 g/L)	168 mg (2.8 mM)	N/A	[27]
*E. coli*	GE (Cit)	Glu	3.5 g/L (58.2 mM)	N/A	[25]
*E. coli*	GE (Thr)	Tryptone (10 g/L)	1.0 g/L (16.6 mM)	N/A	[44]
*E. coli*	GE (1,2-PD)	Glu	1.0 g/L (16.6 mM)	N/A	[28]

## Supplementary Materials

The following are available online at https://www.mdpi.com/2076-2607/8/6/945/s1, Figure S1: Effect on exogenously added acetate on glucose fermentation (20 mM) by *Thermoanaerobacter* strain AK152; Figure S2: Effect on exogenously added 1-butyric acid on glucose fermentation (20 mM) by *Thermoanaerobacter* strain AK152; Figure S3: ^13^C NMR spectra of glucose fermentations (20 mM) by *Thermoanaerobacter* strain AK152 containing added ^13^C1 acetate (A), ^13^C1 propionate (B), ^13^C1 butyrate (C) at a final concentration of 20 mM; Table S1: End product profiles for fermentation of selected substrates by *Thermoanaerobacter* strain AK152 after 5 days of fermentation; Table S2: API ZYM data for *Thermoanaerobacter* strain AK152 and *T. thermohydrosulfuricus*; Table S3: Influence of initial 1-propionate on reduction to 1-propanol during glucose (20 mM) fermentation by *Thermoanaerobacter* strain AK152 after 5 days of fermentation; Supplementary **Table S4**: Influence of initial pH on reduction of 1-propionate to 1-propanol during glucose (20 mM) fermentation by *Thermoanaerobacter* strain AK152 after 5 days of fermentation.
