# Supplementary material for: Biotransformation of Carboxylic Acids to Alcohols: Characterization of Thermoanaerobacter Strain AK152 and 1-Propanol Production via Propionate Reduction
Source: Microorganisms. 2020 Jun 23;8(6):945. doi: 10.3390/microorganisms8060945 (PMC7356315; doi:10.3390/microorganisms8060945)
Supplement: Supplementary file 1 [file microorganisms-08-00945-s001.zip › microorganisms-829610-supplementary.docx]

Supplemental Material

**Biotransformation of Carboxylic Acids to Alcohols: Characterization of *Thermoanaerobacter* strain AK152 and 1-propanol production via propionate reduction**

Sean Michael Scully, Johann Orlygsson^#^

University of Akureyri, Faculty of Natural Resource Sciences, Borgir, Nordurslod 2, 600 Akureyri, Iceland.

^#^To whom correspondence should be addressed; jorlygs@unak.is

E-mail address for Sean Michael Scully is [scully@unak.is](mailto:scully@unak.is)

**List of Figures**

**Figure S1** – Effect on exogenously added acetate on glucose fermentation (20 mM) by *Thermoanaerobacter* strain AK152 (65°C, pH 7.0). A – 20 mM acetate, B – 50 mM acetate, C – 100 mM acetate

**Figure S2** – Effect on exogenously added 1-butyric acid on glucose fermentation (20 mM) by *Thermoanaerobacter* strain AK152 (65°C, pH 7.0). A – 20 mM 1-butyrate, B – 50 mM 1-butyrate, C – 100 mM 1-butyrate

**Figure S3** – ^13^C NMR spectra after 5 days of glucose fermentations (20 mM) by *Thermoanaerobacter* strain AK152 (65°C, pH 7.0) containing added ^13^C1 acetate (A) and ^13^C1 butyrate (B) at a final concentration of 20 mM.

**List of Tables**

**Table S1** – End product profiles for fermentation of selected substrates by *Thermoanaerobacter* strain AK152 after 5 days of fermentation (65°C, pH 7.0). The substrate concentration was 20 mM with the exception disaccharides (10 mM) and polymeric substrates for which 2% w/v was used. Data represents average of triplicate ± standard deviation

**Table S2** – API ZYM data for *Thermoanaerobacter* strain AK152 and *T. thermohydrosulfuricus*

**Table S3** – Influence of initial 1-propionate on reduction to 1-propanol during glucose (20 mM) fermentation by *Thermoanaerobacter* strain AK152 after 5 days of fermentation (65°C, pH 7.0). Data represents average of triplicate ± standard deviation

**Table S4** – Influence of initial pH on reduction of 1-propionate to 1-propanol during glucose (20 mM) fermentation by *Thermoanaerobacter* strain AK152 after 5 days of fermentation (65°C). Data represents average of triplicate ± standard deviation

**Figure S1** – Effect on exogenously added acetate on glucose fermentation (20 mM) by *Thermoanaerobacter* strain AK152 (65°C, pH 7.0). A – 20 mM acetate, B – 50 mM acetate, C – 100 mM acetate


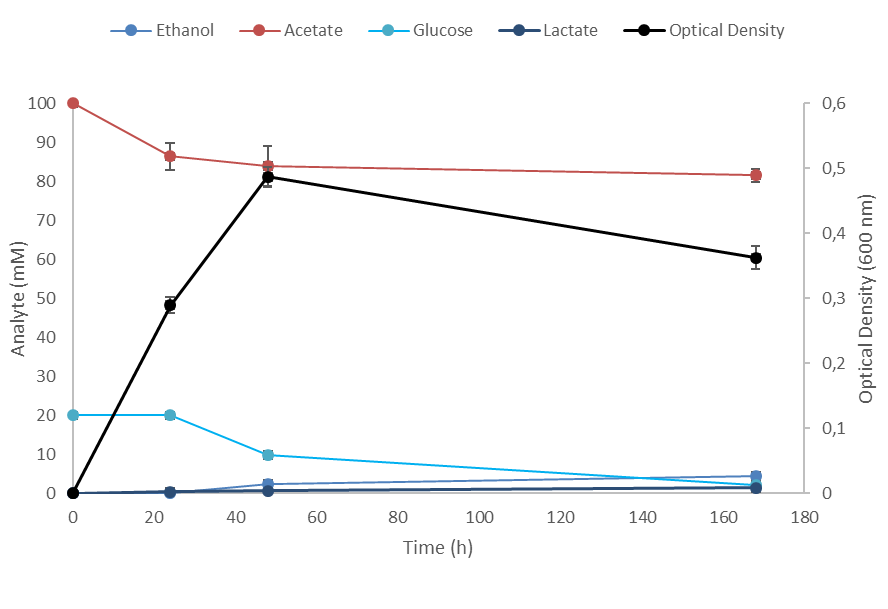


**C**

**Figure S2** – Effect on exogenously added 1*-*butyric acid on glucose fermentation (20 mM) by *Thermoanaerobacter* strain AK152 (65°C, pH 7.0). A – 20 mM 1*-*butyrate, B – 50 mM 1*-*butyrate, C – 100 mM 1*-*butyrate

**Figure S3** – ^13^C NMR spectra after 5 days of glucose fermentations (20 mM) by *Thermoanaerobacter* strain AK152 (65°C, pH 7.0) containing added ^13^C1 acetate (A) and ^13^C1 butyrate (B) at a final concentration of 20 mM.


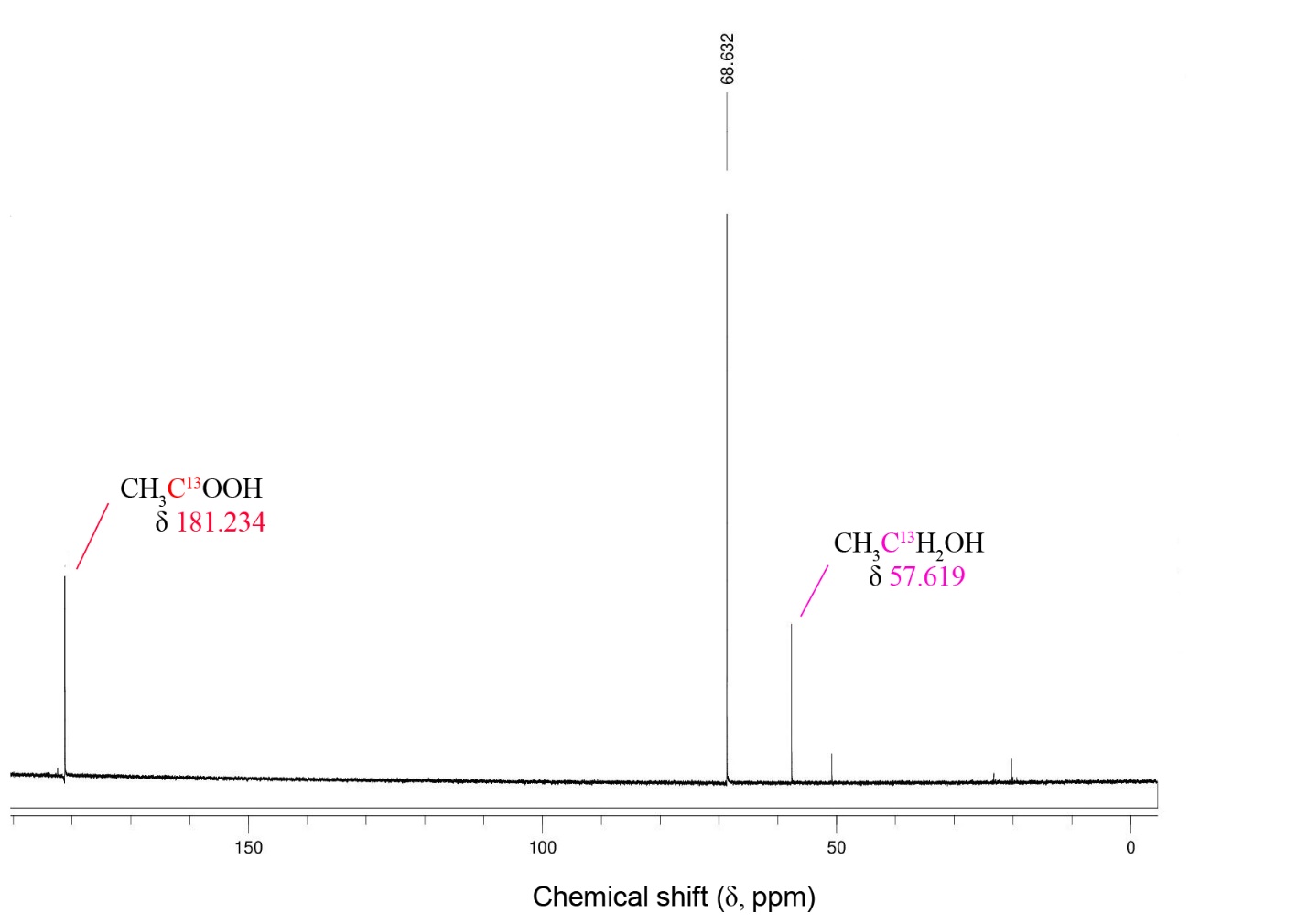


**A**


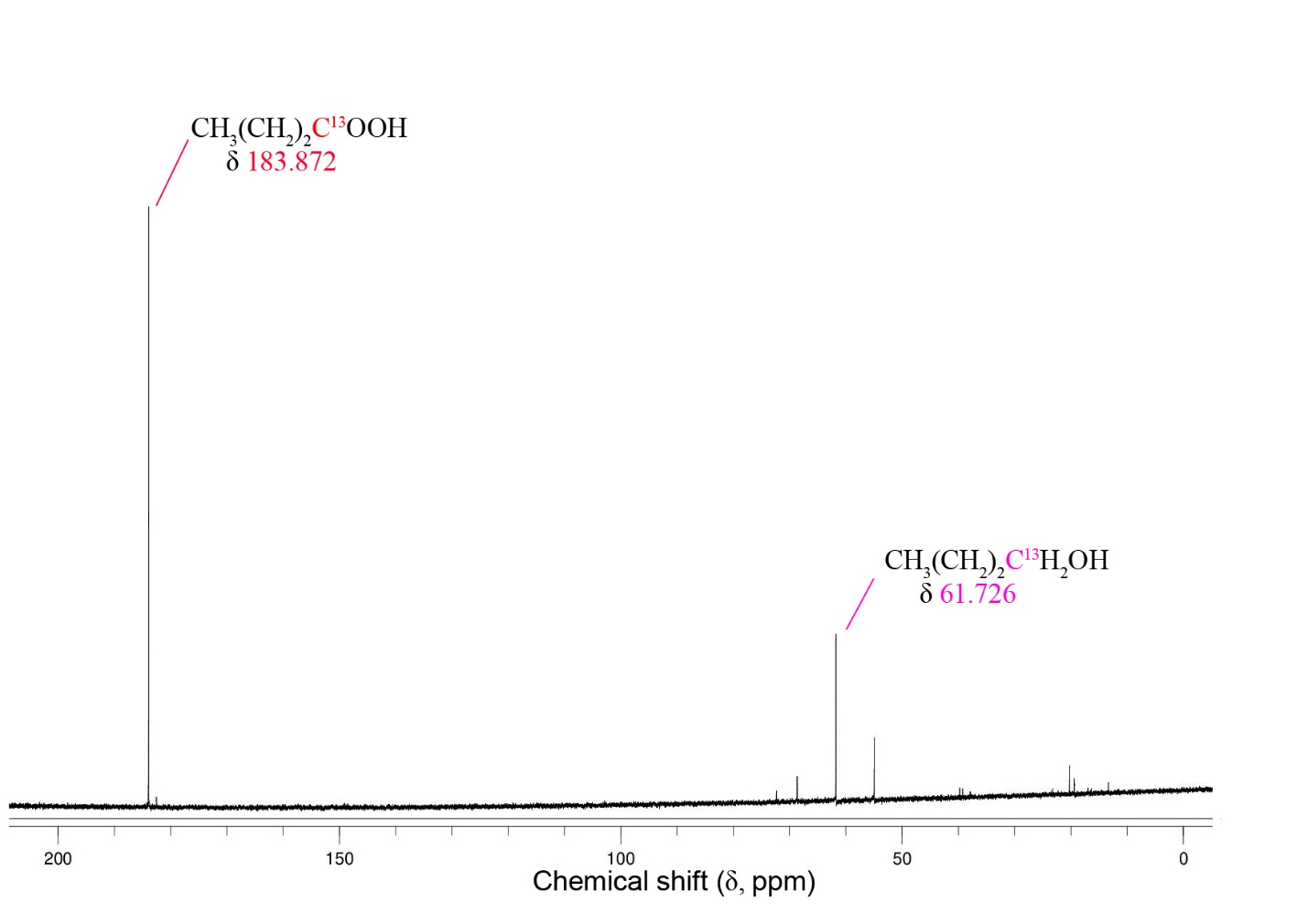


**B**

**Table S1** – End product profiles for fermentation of selected substrates by *Thermoanaerobacter* strain AK152 after 5 days of fermentation (65°C, pH 7.0). The substrate concentration was 20 mM with the exception disaccharides (10 mM) and polymeric substrates for which 2% w/v was used. Data represents average of triplicate ± standard deviation.

|  |  | **Analyte (mM)** | | | |  |  |
| --- | --- | --- | --- | --- | --- | --- | --- |
| **Substrate** |  | **Ethanol** | **Acetate** | **Lactate** | **Hydrogen** | **Optical Density** (600 nm) | **Carbon balance** (%) |
| Control (YE only) |  | 0.14±0.02 | 0.19±0.01 | 0.50±0.07 | 0.00±0.00 | 0.08±0.02 | ND |
| D-Glucose |  | 34.48±0.16 | 3.35±0.01 | 0.72±0.17 | 2.71±0.34 | 0.34±0.03 | 96.39 |
| D-Fructose |  | 29.91±0.69 | 3.84±0.00 | 1.05±0.06 | 3.15±0.03 | 0.29±0.04 | 87.00 |
| D-Galactose |  | 32.03±0.01 | 3.96±0.17 | 0.84±0.10 | 3.05±0.10 | 0.28±0.02 | 92.07 |
| D-Mannose |  | 29.42±0.31 | 3.65±0.03 | 0.93±0.13 | 3.23±0.15 | 0.28±0.01 | 84.99 |
| D-Arabinose |  | 1.34±0.04 | 2.15±0.02 | 0.05±0.00 | 1.06±0.00 | 0.11±0.01 | 8.83 |
| D-Xylose |  | 27.37±2.00 | 4.81±0.34 | 2.31±0.06 | 3.52±0.03 | 0.29±0.03 | 86.23 |
| L-Rhamnose |  | 1.81±0.23 | 1.65±0.15 | 0.05±0.00 | 1.10±0.08 | 0.12±0.01 | 8.77 |
| L-Fucose |  | 1.51±0.05 | 1.31±0.21 | 0.05±0.00 | 0.91±0.04 | 0.08±0.00 | 7.18 |
| Lactose |  | 36.21±1.06 | 5.22±0.51 | 1.03±0.24 | 4.91±0.08 | 0.34±0.04 | 106.14 |
| Maltose |  | 39.11±2.35 | 3.29±0.58 | 1.15±0.19 | 3.23±1.27 | 0.33±0.01 | 108.88 |
| Trehalose |  | 36.07±4.55 | 4.54±0.04 | 0.80±0.11 | 3.92±0.07 | 0.31±0.02 | 103.52 |
| Cellobiose |  | 35.81±5.41 | 6.75±0.00 | 1.03±0.18 | 5.65±0.13 | 0.30±0.04 | 108.96 |
| Sucrose |  | 2.63±0.14 | 1.86±0.21 | 0.31±0.04 | 1.34±0.04 | 0.10±0.00 | 12.00 |
| Raffiose |  | 2.30±0.22 | 2.06±0.28 | 0.48±0.05 | 1.35±0.02 | 0.30±0.05 | 12.09 |
| Glycerol |  | 2.69±0.11 | 1.80±0.09 | 0.30±0.07 | 0.85±0.06 | 0.10±0.00 | 11.97 |
| Pyruvate |  | 6.04±0.34 | 14.60±0.39 | 0.94±0.13 | 3.57±0.96 | 0.15±0.02 | 53.95 |
| Xylan (oat) |  | 4.59±0.06 | 4.47±0.19 | 1.82±0.16 | 1.53±0.04 | 0.21±0.04 | ND |
| Starch (potato) |  | 20.72±0.80 | 3.89±0.11 | 1.54±0.20 | 3.01±0.07 | 0.37±0.03 | ND |
| Carboxymethyl cellulose (CMC) |  | 2.21±0.10 | 2.51±0.01 | 0.05±0.00 | 1.42±0.04 | 0.05±0.00 | ND |
| Cellulose |  | 2.56±0.05 | 2.72±0.17 | 0.05±0.00 | 1.25±0.25 | 0.04±0.01 | ND |
| Avicel |  | 2.10±0.05 | 2.37±0.01 | 0.05±0.00 | 0.97±0.19 | 0.06±0.02 | ND |
| Serine |  | 3.70±0.23 | 17.73±1.12 | 0.00±0.00 | 6.95±1.12 | 0.21±0.03 | 53.56 |
| Theronine |  | 2.30±0.10 | 2.39±0.26 | 0.00±0.00 | 1.47±0.22 | 0.11±0.02 | 11.72 |

**Table S2** – API ZYM data for *Thermoanaerobacter* strain AK152 and *T. thermohydrosulfuricus.*

|  | Alkaline phosphatase | Esterase (C4) | Lipase esterase (C8) | Lipase (C14) | Leucine arylamidase | Valine arylamidase | Cysteine arylamidase | Trypsin | α-Chymotrypsin | Acid phosphatase | Naphthol-AS-BI-phosophohydrolase | α-Galactosidase | β-Galactosidase | β-Glucuronidase | α-Glucosidase | β-Glucosidase | *N*-Acetyl-β-glucosaminidase | α-Mannosidase | α-Fucosidase |
| --- | --- | --- | --- | --- | --- | --- | --- | --- | --- | --- | --- | --- | --- | --- | --- | --- | --- | --- | --- |
| *Thermoanaerobacter* strain AK152 | - | **+** | - | - | - | - | - | - | - | **+** | **+** | - | - | - | - | - | **(+)** | - | - |
| *T. thermohydrosulfuricus* (DSM 567) | - | **+** | **+** | - | - | - | - | - | - | **+** | **+** | - | - | - | - | **+** | **+** | - | - |

**Table S3** – Influence of initial 1-propionate on reduction to 1-propanol during glucose (20 mM) fermentation by *Thermoanaerobacter* strain AK152 after 5 days of fermentation (65°C, pH 7.0). Data represents average of triplicate ± standard deviation.

|  |  | **Analyte (mM)** | | | | | | |  |  |  |  | |  |
| --- | --- | --- | --- | --- | --- | --- | --- | --- | --- | --- | --- | --- | --- | --- |
| **Initial propionate  concentration (mM)** |  | **Ethanol** | **1-Propanol** | **Acetate** | **Lactate** | **1-propionate** | **Hydrogen** | **Glucose  degradation (%)** | | **RCOOH  conversion (%)** | **Optical Density  (600 nm)** | | **Carbon balance (%)** | |
| Control (YE only) |  | 0.45±0.15 | 0.00±0.00 | 4.37±0.73 | 0.00±0.00 | 0.00±0.00 | 4.74±1.23 | ND | | ND | 0.10±0.00 | | ND | |
| 0 |  | 25.32±0.37 | 0.00±0.00 | 15.58±0.23 | 4.51±0.53 | 0.00±0.00 | 16.51±0.94 | 100 | | 0.0 | 0.25±0.03 | | 88.5 | |
| 10 |  | 14.64±1.45 | 2.44±0.72 | 15.58±1.65 | 4.24±0.61 | 5.98±0.85 | 18.69±1.84 | 97.3 | | 24.0 | 0.27±0.01 | | 85.7 | |
| 20 |  | 10.79±0.09 | 8.37±0.08 | 15.55±0.71 | 4.73±0.41 | 13.11±2.86 | 23.14±1.16 | 98.7 | | 41.9 | 0.29±0.02 | | 91.1 | |
| 40 |  | 10.24±0.29 | 7.24±0.58 | 17.63±0.98 | 4.89±0.51 | 30.45±1.18 | 22.38±2.10 | 99.17 | | 18.1 | 0.25±0.03 | | 90.4 | |
| 60 |  | 8.21±0.11 | 2.82±0.10 | 19.51±2.14 | 4.69±0.34 | 52.37±8.91 | 21.78±1.50 | 96.8 | | 4.7 | 0.26±0.03 | | 85.7 | |
| 80 |  | 7.37±0.29 | 2.48±0.39 | 17.58±1.13 | 6.24±1.66 | 59.85±12.96 | 17.19±3.44 | 90.9 | | 3.1 | 0.23±0.02 | | 77.1 | |

**Table S4** – Influence of initial pH on reduction of 1-propionate to 1-propanol during glucose (20 mM) fermentation by *Thermoanaerobacter* strain AK152 after 5 days of fermentation (65°C). Data represents average of triplicate ± standard deviation.

|  |  | **Analyte (mM)** | | | | | | |  |  |  |  | |
| --- | --- | --- | --- | --- | --- | --- | --- | --- | --- | --- | --- | --- | --- |
| **Initial pH** |  | **Ethanol** | **1-Propanol** | **Acetate** | **Lactate** | **1-propionate** | **Hydrogen** | **Glucose  degradation (%)** | | **RCOOH  conversion (%)** | **Optical Density  (600 nm)** | | **Carbon  balance (%)** |
| 5.0 |  | 12.23±0.18 | 6.07±0.17 | 12.31±0.37 | 1.08±0.08 | 12.21±0.23 | 2.01±0.24 | 70.8 | | 30.4 | 0.17±0.02 | | 90.9 |
| 5.5 |  | 15.31±0.91 | 7.31±0.37 | 14.71±0.31 | 2.21±0.10 | 12.51±0.62 | 13.53±0.67 | 88.2 | | 36.6 | 0.24±0.08 | | 91.0 |
| 6.0 |  | 17.23±0.97 | 9.21±0.51 | 12.31±0.71 | 2.47±0.18 | 10.09±0.51 | 12.27±0.47 | 95.3 | | 46.1 | 0.29±0.20 | | 84.5 |
| 6.5 |  | 24.33±0.54 | 11.45±0.20 | 13.78±0.42 | 2.54±0.29 | 8.21±0.60 | 13.23±0.55 | 100.0 | | 57.3 | 0.25±0.11 | | 96.3 |
| 7.0 |  | 22.00±0.43 | 8.67±0.32 | 13.71±0.27 | 2.68±0.21 | 11.34±0.36 | 12.17±0.85 | 100.0 | | 43.4 | 0.36±0.03 | | 92.9 |
| 7.5 |  | 25.94±0.67 | 8.38±0.21 | 13.34±0.21 | 1.98±0.34 | 10.99±0.47 | 12.37±0.92 | 100.0 | | 41.9 | 0.34±0.03 | | 97.8 |
| 8.0 |  | 23.32±0.23 | 7.91±0.30 | 12.89±0.42 | 2.21±0.33 | 12.30±0.44 | 11.81±0.59 | 96.5 | | 39.6 | 0.32±0.04 | | 96.4 |
| 8.5 |  | 18.21±0.5 | 5.81±0.17 | 6.89±0.65 | 2.33±0.15 | 12.87±0.38 | 7.38±0.41 | 90.6 | | 29.1 | 0.28±0.02 | | 79.3 |
